# Lung aeration in experimental malaria-associated acute respiratory distress syndrome by SPECT/CT analysis

**DOI:** 10.1371/journal.pone.0233864

**Published:** 2020-05-29

**Authors:** Thatyane de Castro Quirino, Luana dos Santos Ortolan, Michelle Klein Sercundes, Claudio Romero Farias Marinho, Walter Miguel Turato, Sabrina Epiphanio

**Affiliations:** 1 Departamento de Análises Clínicas e Toxicológicas, Faculdade de Ciências Farmacêuticas, Universidade de São Paulo, São Paulo, Brasil; 2 Departamento de Imunologia, Instituto de Ciências Biomédicas, Universidade de São Paulo, São Paulo, Brasil; 3 Departamento de Parasitologia, Instituto de Ciências Biomédicas, Universidade de São Paulo, São Paulo, Brasil; 4 Centro de Radiofarmácia, Instituto de Pesquisas Energéticas e Nucleares, São Paulo, Brasil; Instituto Rene Rachou, BRAZIL

## Abstract

Malaria-associated acute respiratory distress syndrome (ARDS) is an inflammatory disease causing alveolar-pulmonary barrier lesion and increased vascular permeability characterized by severe hypoxemia. Computed tomography (CT), among other imaging techniques, allows the morphological and quantitative identification of lung lesions during ARDS. This study aims to identify the onset of malaria-associated ARDS development in an experimental model by imaging diagnosis. Our results demonstrated that ARDS-developing mice presented decreased gaseous exchange and pulmonary insufficiency, as shown by the SPECT/CT technique. The pulmonary aeration disturbance in ARDS-developing mice on the 5^th^ day post infection was characterized by aerated tissues decrease and nonaerated tissue accumulation, demonstrating increased vascular permeability and pleural effusion. The SPECT/CT technique allowed the early diagnosis in the experimental model, as well as the identification of the pulmonary aeration. Notwithstanding, despite the fact that this study contributes to better understand lung lesions during malaria-associated ARDS, further imaging studies are needed.

## Introduction

Malaria-associated acute respiratory distress syndrome (ARDS) is an acute inflammatory pulmonary lesion characterized by severe hypoxia [1,2], leading to vascular permeability increase [3,4], which causes pleural effusion and lung weight enlargement [5], and also reduces aerated lung tissue resulting in high mortality (30–40%) in intensive care units [6–8]. Seven species of Plasmodium, etiological malaria agents, have been identified as capable of infecting humans [9,10]. However, infections by *Plasmodium falciparum* and *Plasmodium vivax* showed 95% prevalence of malaria in the world [11]. In spite of the progress in its prophylaxis and treatment [12–14], malaria is still considered an important public health problem [15]. World Malaria Report published in 2019 showed an increase in malaria cases, stating 228 million human infections and 405 thousand deaths in 2018 [16].

Imaging procedures such as chest X-ray and computed tomography (CT) [17], gold standard techniques in ARDS identification [18], along with others, such as electrical impedance, positron emission tomography (PET) [19] and ultrasonography [20] are fundamental for diagnosis and analysis of lung function during ARDS development [21,22]. Through X-ray technique, it is possible to observe the opacity of bilateral diffuse infiltrate in humans lungs [23] and unilateral opacification in mice lungs [5]. On the other hand, pulmonary topographic analyzes during ARDS in mice demonstrate that the respiratory capacity has been decrease with normally aerated tissue reduction and increase nonaerated tissues. In addition, those lungs can be associated with atelectasis [24], ground-glass attenuation, consolidation in ARDS acute phase and reticulation attenuation with fibrosis in late phase [17].

The densitometry determines the lung compartments, according to X-ray attenuation values, and shows absence and presence of air inside the lungs, characterized by a voxel -700 Hounsfield Units (HU) which represents 70% gas and 30% tissue or water [25,26]. In addition, the lungs are characterized by hyperexpanded (-1000 to -900HU) compartments, normally aerated (-900 to -500HU), poorly aerated (-500 to -100HU) and nonaerated (0 to 100HU) [27–29]. Perfusion studies are critical to assess lung function and determine the blood flow available for gas exchange [30,31]. The main radionuclide used for single-photon emission computed tomography (SPECT) for diagnostic target is the metastable technetium 99 (^99m^Tc) [32], a low energy gamma radiation emitter (140keV), which confers reduced cost and physical half- life of approximately six hours, making it possible to acquire high resolution scintigraphic images in gamma cameras [33,34]. The albumin macroaggregate (MAA) conjugated to ^99m^Tc is a radiopharmaceutical with affinity for the lung and it is used in clinical diagnosis in nuclear medicine [35,36]. Furthermore, a better understanding of the pathogenesis and also the validation of new tools such as SPECT/CT studies are necessary for malaria-associated ARDS early diagnosis.

## Materials and methods

### Mice and ethics statement

DBA/2 male mice, 6–8 weeks old, specific pathogens free (SPF), were maintained in 12/12h light/dark cycle and acclimatized for 1 week before the experiments. The maximum of five mice per cage were housed in a standard polycarbonate cage with wood shavings bedding, an SPF animal house. We also attempted to reduce the stress of individual housing by environmental enrichment with small play tunnels. Mice had *ad libitum* access to food and water. The mice were initially imported from The Jackson Laboratory (USA) and the colonies were maintained in the Animal House of the Faculty of Pharmaceutical Sciences of the University of São Paulo (FCF/USP).

The experiments were performed according to the ethical guidelines established by the National Council for Control of Animal Experimentation (CONCEA: Conselho Nacional de Controle de Experimentação Animal) and Brazilian Federal Law nº 11.794 and approved by Animal Health Committee of the Biomedical Sciences Institute of the University of São Paulo (CEUA-ICB/USP, protocol number 05/2017). The ARRIVE guidelines recommendations are followed in this study.

### *Plasmodium berghei* ANKA infection, parasitemia and euthanasia

Ten to twelve male DBA/2 mice per group were injected with 10^6^ *P*. *berghei* ANKA (clone 1.49L)-infected red blood cells (iRBCs), kindly provided by the laboratory of Dra. Maria Mota from the institute of Molecular Medicine (IMM) in Portugal, by intraperitoneal route, as previously described [5,37]. Parasitemia was expressed as percentage of iRBCs and was determined by Giemsa staining. After infection, mice clinical signs were evaluated twice a day. Mortality was monitored from the 5^th^ to 17^th^ day post infection (dpi). The experimental time course to was reduced to 17 days after infection, compared with other experiments of our model previously published [5,37–39] to shorten any discomfort, pain or suffering that *Plasmodium berghei* infection may cause.

The euthanasia of DBA/2 mice was executed on the 17^th^ dpi, using ketamine (150 mg/kg)/xylazine (15 mg/kg). Consciousness was checked by testing the pedal reflex, heartbeats, and breathing movements. In addition, the mice were humanely euthanized (end point) once they showed the following clinical signs: lethargy, hypothermia, and/or difficulty to breathe [measure in a whole-body plethysmography chamber (WBP, Buxco Electronics, Wilmington, North Carolina, USA)]. If these parameters were not properly registered, failing to achieve basal levels, it meant the mice were having severe breathing problems and consequently, euthanized.

### Image protocol

The experiments were performed by using PET/SPECT/CT Albira 5.0 scanner system (Carestream Molecular Imaging, Woodbridge, CT, USA). *Plasmodium berghei* ANKA-infected (5^th^ dpi, 7^th^ dpi, 14^th^ dpi) and healthy DBA/2 male mice were anesthetized intramuscularly or subcutaneously with Ketamine (100mg/kg) and Xilazine (10mg/kg). They were placed in prona position for images acquisition. Pulmonary perfusion was performed with 50–100μl of 258μCi of ^99m^Tc-MAA (Pul-Tec, IPEN, São Paulo, Brazil) by intravenous route. The SPECT/CT acquisition was performed using single pinhole collimators in a 50 mm field view, being the energy and X-ray current characteristics 35 kVp and 400 μA, respectively. The simplified protocol of the methodology sequence for image acquisition is described in S1 Fig.

### 3D reconstruction of SPECT/CT images

Initial SPECT and CT data were reconstructed with Ordered-Subsets Expectation Maximization (OSEM) and Filtered Back Projection (FBP) algorithms by using Albira suite reconstruction and μPMOD version 5.0 (PMOD technologies, Zurich, Switzerland) softwares, in order to obtain images with spatial resolution of 1.8 mm and 0.125 mm, respectively. Mask and cropped tools were applied in the initial files so that lungs were segmented in different aeration threshold. A total of 1,000 projections/mice for CT and 60 projections/mice for SPECT were performed at 30 second/projection duration and approximately 1 hour and 20 min for total acquisition.

### Quantitative analysis of SPECT/CT images

The SPECT and CT images were quantified using the μPMOD version 5.0 program (PMOD technologies, Zurich, Switzerland). Briefly, each CT image was segmented according to HU values. For each region of interest (ROI), in right and left lungs, 3D segmentation was performed, in which the voxels number was calculated, evaluating their respective mass and volume, using 25/25 HUs as the initial threshold [27,40]. In this study, we determined the pulmonary compartments as follows: hyperexpanded (-1000 HU to -900 HU), normally aerated (-900 HU to -500 HU), poorly aerated (-500 to -100 HU) and nonaerated (-100 to +100 HU). For pulmonary perfusion, ^99m^Tc-MAA activity analysis was used and the images were processed using the 3D Slicer version 4.8 (3DSlicer technologies, Harvard, USA) employing the Segment Editor to select the ROI and the threshold tool.

### Respiratory parameters quantification

Mice respiratory parameters (respiratory rate, enhanced pause (Penh), inspiratory and expiratory time, ventilation volume and tidal volume) were collected and quantified using unrestrained whole-body plestimography chambers (Buxco Eletronics, Harvard, USA) and FinePointe software, as previously described [39] on the 5^th^ and 7^th^ dpi.

### Statistical analysis

The quantitative variables were presented as mean ± standard deviation (SD), being their normality data verified by Kolmogorov or Shapiro-Wilk tests. Nonparametric data were compared using Mann-Whitney test (2 groups) or Kruskal-Wallis (3 groups) followed by Dunn's post-hoc test. In the nonparametric correlation analysis, the Spearman test (r) was used, followed by the linear regression test. The differences between the groups were considered significant when *p* <0.05. All data were analyzed using GraphPad Prism 5.0 software.

## Results

### Malaria-associated acute respiratory distress syndrome characterization in a murine model

**Fig 1:**
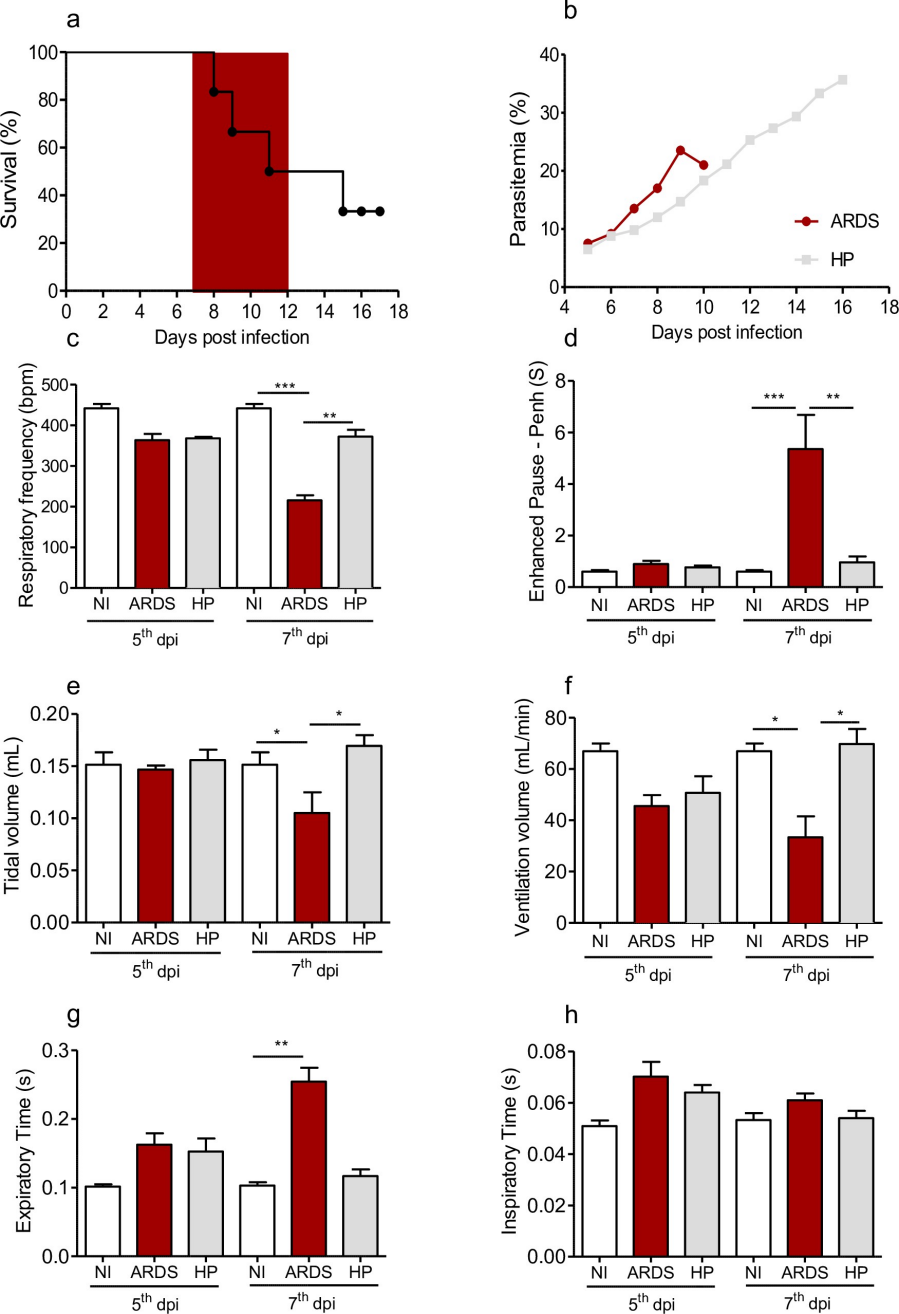
Evaluation of survival, parasitemia and respiratory parameters during malaria-associated ARDS in experimental model. (a) Survival curve, (b) parasitemia, (c) respiratory frequency, (d) enhanced pause (Penh), (e) tidal volume, (f) ventilation volume, (g) exhalation time and (h) inspiration time in *Plasmodium berghei*-infected DBA/2 mice on the 5^th^ and 7^th^ days post infection (dpi). The ARDS-developing mice died between 7^th^ and 12^th^ dpi, identified by gray band (a). Data presented as mean and standard error. Kruskal-Wallis Test, * p <0.01, ** p <0.001, ** p <0.0001, n = 7–12 mice/ experiment. Representative figures from 2–3 independent experiments NI: Non-Infected mice, ARDS: Acute Respiratory Distress Syndrome, HP: Hyperparasitemia.

During plasmodium infections, survival, parasitemia and respiratory parameters (respiratory rate, Penh, inspiratory and expiratory time, ventilation volume and tidal volume) were analyzed in order to identify the mice developing ARDS or Hyperparasitemia (HP) (Fig 1). The ARDS-developing mice, which died between 7^th^ and 12^th^ dpi (Fig 1A), showed pulmonary edema and hemorrhage as previously reported [4,5]. On the other hand, HP-developing mice which died after 13^th^ dpi, showed pale and grayish lungs due to malarial pigment accumulation and anemia, as related before [5]. The parasitemia showed no significant differences between ARDS-developing and HP-developing mice, on the 5^th^ dpi, but it was higher in the former, on the 7^th^ dpi (p<0.05) (Fig 1B). In addition, ARDS-developing mice, on the 7^th^ dpi, showed decreased respiratory frequency (Fig 1C), increased enhanced pause (Fig 1D), diminished tidal volume (Fig 1E), reduced ventilation volume (Fig 1F), prolonged expiration time (Fig 1G), but did not show significant differences in inspiration time (Fig 1H) when compared to those non-infected or HP-developing mice.

### Imaging diagnosis of experimental malaria-associated acute respiratory distress syndrome analyzed by computed tomography

**Fig 2:**
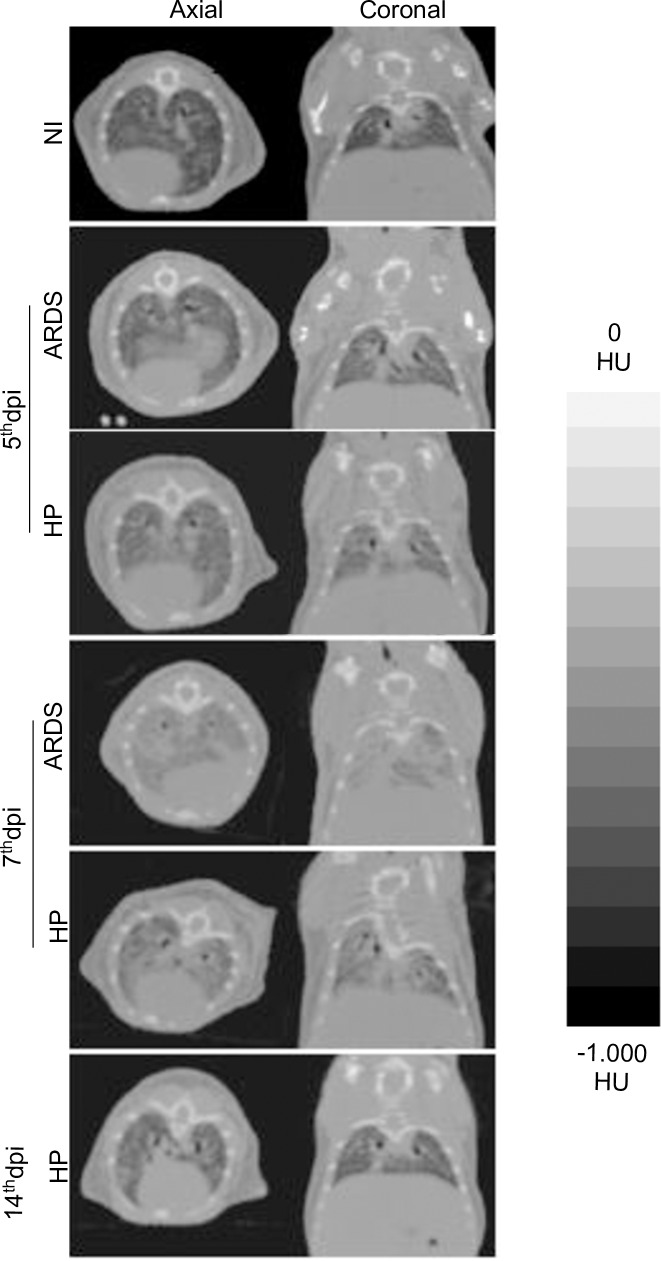
Morphological evaluation during the development malaria-associated ARDS by computed tomography. Representative topographic images of *P*. *berghei* ANKA-infected DBA/2 mice. The scale by Hounsfield Units 0 HU (Water Density) to -1,000 HU (Air Density). Representative figures from 2–4 independent experiments, n = 7–14 animals/experiment. NI: Non-Infected mice, ARDS: Acute Respiratory Distress Syndrome, HP: Hyperparasitemia; dpi: days post infection.

The qualitative analysis of pulmonary aeration during experimental ARDS (Fig 2), demonstrated that ARDS-developing mice, on the 7^th^ dpi, had a marked decrease in aeration extensive areas compatible with consolidation associated with diffuse opacification in all topographic cuts, when compared to HP-developing, ARDS-developing (on the 5^th^ dpi) and non-infected mice, which had healthy lungs and intense aeration. HP-developing mice showed decreased aerated tissues and marked opacification, on the 7^th^ dpi, when compared with the 5^th^ dpi and with non-infected mice.

However, HP-developing mice showed increased aerated lung tissue, on the 14^th^ dpi, when compared with the 7^th^ dpi. The predominance of ground-glass opacification pulmonary lesions in ARDS-developing mice, on the 7^th^ dpi, demonstrating alveolar collapse followed by a decrease in respiratory capacity was observed.

### Experimental malaria promotes lung aeration reduction analyzed by computed tomography

**Fig 3:**
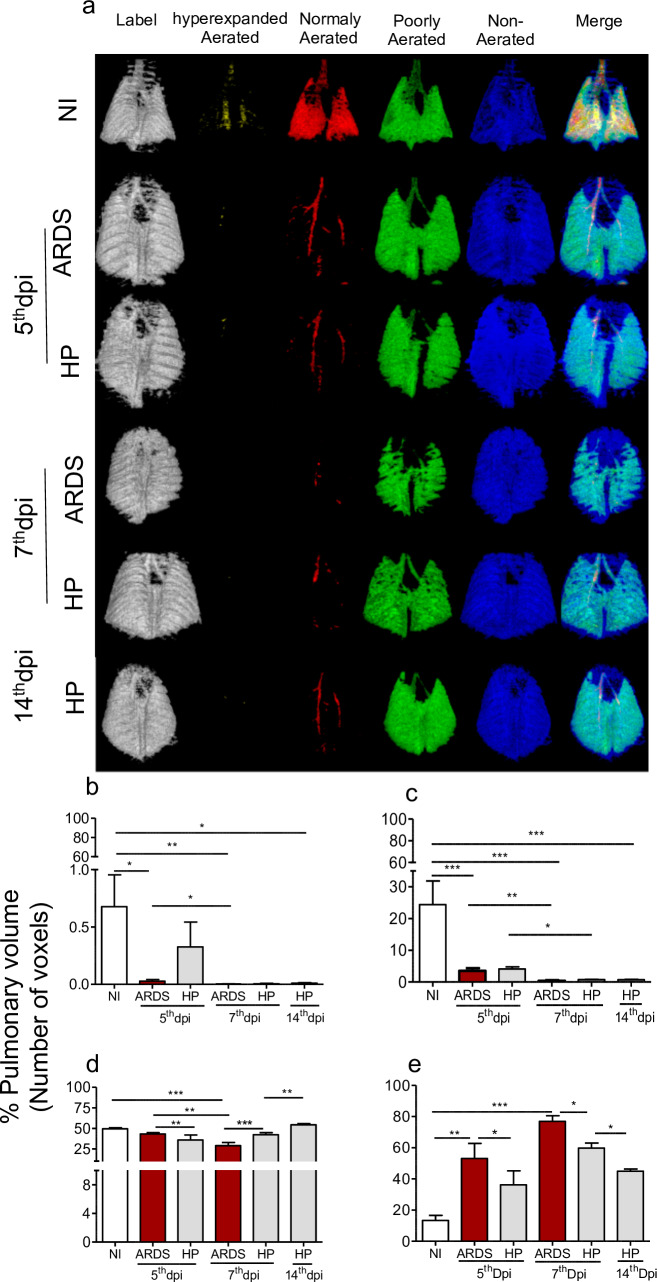
Malaria-associated ARDS decreases pulmonary aeration and promotes atelectasis as shown in computed tomography. (a) Coronary posterior plane 3D pulmonary segmentation; Gray: Label CT, Yellow: Hyperexpanded, Red: Normally Aerated, Green: Poorly Aerated, Blue: Non-Aerated, Overlap: Merge. (b-e) Quantification of pulmonary aeration by computed tomography in DBA/2 mice after 5^th^, 7^th^, and 14^th^ days of *Plasmodium berghei* ANKA infection. (b) hyperexpanded aerated tissues; (c) Normally aerated tissues; (d) Poorly aerated tissues; (e) Non-aerated tissues; Representative images of 2 independent experiments, n = 8-12/experiment. Kruskal-Wallis. * p <0.05, ** p <0.01, ** p <0.001. NI: Non-Infected, ARDS: Acute respiratory distress syndrome, HP: Hyperparasitemia, dpi: days post infection.

In the 3D reconstructions and quantification of the pulmonary aeration (Fig 3), we could observe small areas of hyperinflation in the coronal topographies of non-infected and *P*. *berghei* ANKA-infection mice promoted an evident reduction in hyperexpanded tissues in DBA/2 mice, when compared to those non-infected, during all days of analyzes. However, there was no significant difference between ARDS-developing and HP-developing, on the 5^th^ and 7^th^ dpi (Fig 3A and 3B).

Non-infected mice presented higher values of normally aerated tissue when compared to *P*. *berghei* ANKA-infected ones in all the analyzed days (Fig 3A and 3C). Nonetheless, ARDS-developing mice, on the 5^th^ and 7^th^ dpi, showed the lowest values of normally aerated, exhibiting pulmonary commitment (NI vs ARDS on the 5^th^ dpi p <0.001, NI vs ARDS on the 7^th^ dpi p <0.001). No significant differences were observed in pulmonary aeration between ARDS-developing and HP-developing mice on the 5^th^ or 7^th^ dpi (p>0.05). Interestingly, ARDS-developing mice showed a decrease in aerated (hyperexpanded and normally aerated) tissues on the 5^th^ dpi (p <0.01), suggesting that the longitudinal evaluation may allow the detection of ARDS alterations earlier, once the aeration profile of HP-developing did not change on the 5^th^ dpi when compared to those mice on the 7^th^ dpi (p>0.05). On the other hand, on the 14^th^ dpi, the lungs of the HP-developing mice exhibited the most conserved areas, displaying pulmonary lesions reduction (Fig 3A and 3C).

Regarding poorly aerated tissues, HP-developing mice showed a significant decrease when compared to ARDS-developing, on the 5^th^ dpi (p> 0.01), but, on the 7^th^ dpi, ARDS-developing exhibited tissues decrease when compared to HP-developing and non-infected mice (p>0.001). ARDS-developing mice, on the 5^th^ and 7^th^ dpi, did not demonstrate poorly aerated tissue differences when compared to those non-infected. Also, we observed an increase in poorly aerated tissues in HP-developing mice on the 14^th^ dpi when compared to those on the 7^th^ dpi. (p> 0.01) (Fig 3A and 3D).

Non-infected mice showed small areas of non-aerated tissues and, after infection, these regions increased on all analyzed days when compared to controls. ARDS-developing mice showed interesting results regarding nonaerated tissues, which increased when compared to all other mice. On the 7^th^ dpi, ARDS-developing mice presented approximately 80% of non-aerated lung tissues, reaching 4-fold increase compared to non-infected ones, demonstrating increased pulmonary vascular permeability and, consequently, fluid accumulation (edema), hemorrhages and congestion areas. Finally, ARDS-developing mice, on the 5^th^ dpi, presented 2-fold increase of those tissues when compared to the controls, allowing early identification of pulmonary collapse (Fig 3A and 3E).

### Increased non-aerated lung tissue is related to respiratory capacity during experimental malaria-associated ARDS

**Fig 4:**
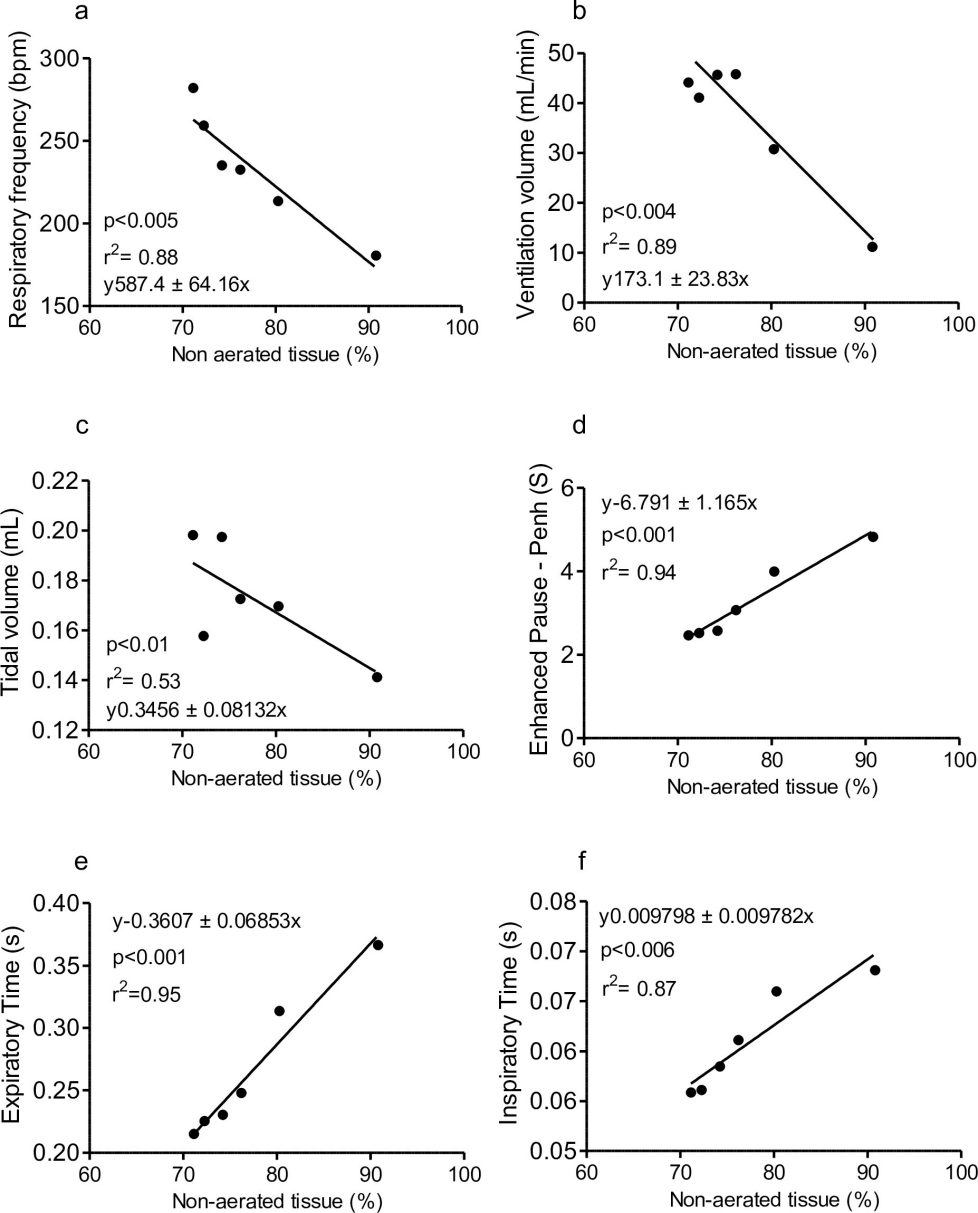
Correlation between non-aerated lung tissue by computed tomography with respiratory parameters during malaria-associated ARDS. (a) Strong negative correlation between decreased respiratory parameters with increased non-aerated CT tissue (p <0.005). (b) Moderate negative correlation between volume per minute of ventilation with increased non-aerated CT tissue. (c) Moderate negative correlation between tidal volume with the increase of non-aerated CT tissue (p <0.01. (d) Strong positive correlation between increased respiratory pause with the presence of non-aerated CT tissue (p <0.001). (e) Strong positive correlation between expiration time with increased non-aerated CT tissue (p <0.001). (f) Strong positive correlation between inspiratory time with increased non-aerated CT tissue (p <0.006). Data presented as mean ± SD. Correlation: Linear Regression (R2) test, n = 6 mice.

On the 5^th^ dpi, there was no correlation between respiratory parameters, as respiratory frequency, increased respiratory pause, inspiratory and expiratory time, ventilation volume and tidal volume, and CT data, because there weren’t differences in these respiratory parameters between non-infected and infected mice. However, performing the linear regression on the 7^th^ dpi, we observed a correlation between these respiratory parameters and an increase in non-aerated tissues (Fig 4). ARDS-developing mice presented a strong negative correlation between decrease of respiratory rate and increase of non-aerated tissue (R^2^ 0.88 p<0.005) (Fig 4A); negative correlation between the decrease of ventilation volume and increase in non-aerated tissues (R^2^ 0.89 p <0.004) (Fig 4B); negative correlation between the decrease of tidal volume and increase of non-aerated tissue (R^2^ 0.53 p <0.01) (Fig 4C); positive correlation between increased enhanced pause and non-aerated tissues (R^2^ 0.94 p <0.001) (Fig 4D) and between increased expiration time and non-aerated tissue (R^2^ 0.95 p <0.001) (Fig 4E); positive correlation between increased inspiratory time and increased atelectasis (R^2^ 0.87 p <0.006) (Fig 4F).

### ARDS-developing mice analyzed by SPECT show decreased pulmonary perfusion

**Fig 5:**
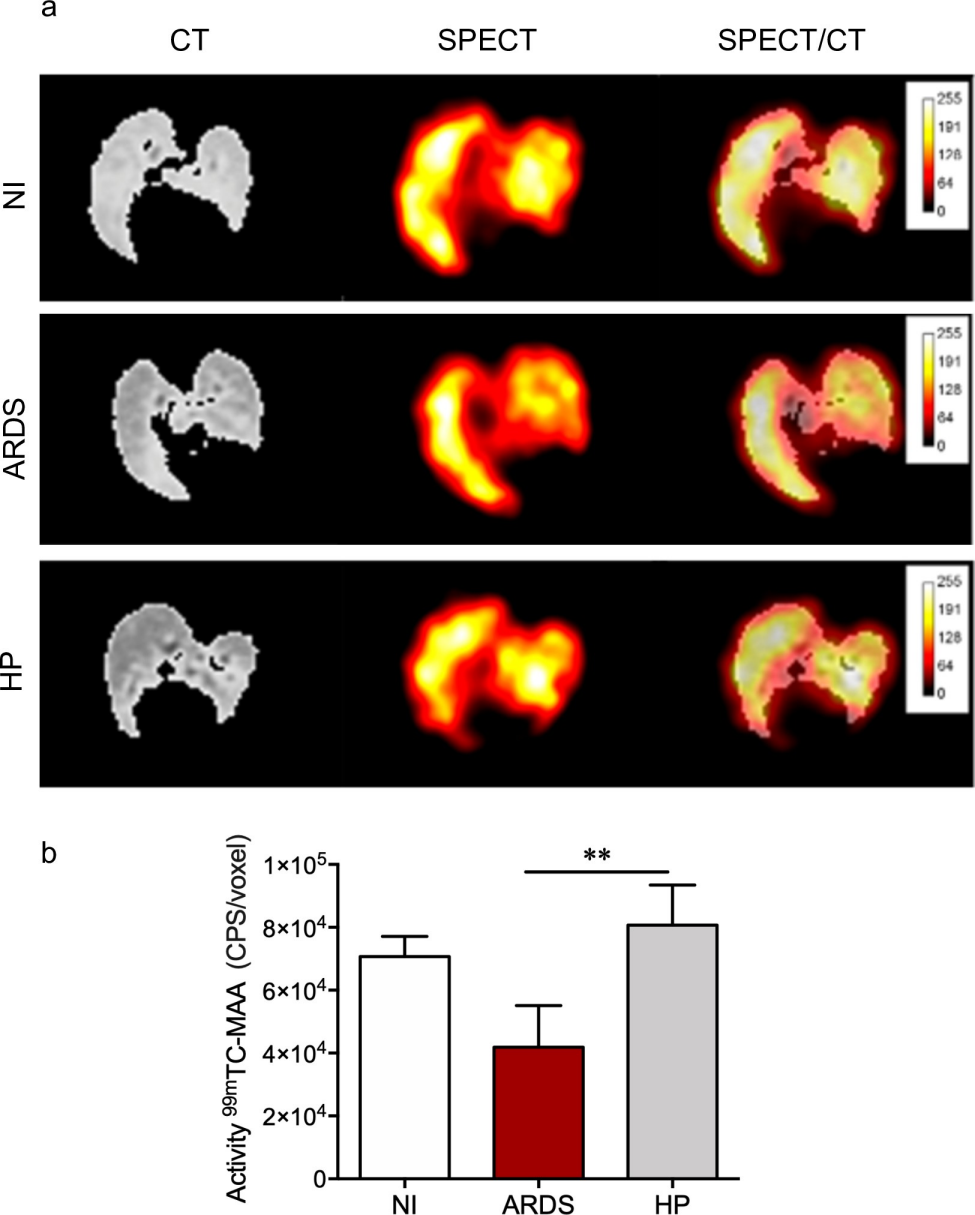
Malaria-associated ARDS promotes decreased ^99m^Tc-MAA activity during pulmonary perfusion. (a) ARDS-developing mice presented absence of left lung perfusion in the coronal plane. (b) Reduced quantification of ^99m^Tc-MAA activity in the lungs of ARDS-developing mice. Data represented as mean ± SD. One Way ANOVA, ** p <0.01. Representative images of 2 isolated experiments, n = 7–10 mice/experiment. CPS/voxel: counts per second per voxel, NI: Noninfected mouse, ARDS: acute respiratory distress syndrome, HP: hyperpasitemia.

Pulmonary function was assessed, and our results showed a decrease of ^99m^Tc-MAA activity during pulmonary perfusion assay in ARDS-developing mice on the 7^th^ dpi (Fig 5A) in all topographic plans, when compared to non-infected and HP-developing mice. In addition, in ARDS-developing mice, we observed a different pattern of blood flow, characterized by marked reduction of perfusion in the left lung and absence of uniform radiopharmaceutical distribution, when compared to non-infected and HP-developing mice (Fig 5B), because these former mice showed no or lower inflammatory process, compared to ARDS-developing mice, demonstrating larger pulmonary distribution of the radiopharmaceutical.

## Discussion

Considering the critical ability to study vital pulmonary processes as well as the understanding of ARDS development, we used a SPECT/CT methodology to measure the respiratory capacity in experimental malaria-associated ARDS using *Plasmodium berghei* ANKA-infected DBA/2 mice. The choice of murine model to this study, malaria-associated ARDS using *Plasmodium berghei* ANKA-infected DBA/2 mice, is due to its high reproducibility and effectiveness, showing also great similarity to ARDS patients [4,5]. Others models of experimental ARDS were reported using *P*. *berghei*-infected C57BL/6 mice [41,42]. However, it is not possible to previously predict which mice will develop ARDS or not, and sometimes, these mice could develop cerebral malaria [43–45].

This preclinical tool can support future investigations into the pathogenesis of ARDS, helping to fill the gap for clinical comprehension of the disease. Changes in respiratory parameters are crucial to identify lung injury in ARDS patients [46]. The reduced respiratory rate and increased pulmonary resistance, due to deficiency in pulmonary oxygenation, makes the ventilatory state monitoring fundamental to prevent the death of the patient [47].

The predictive criteria have been developed to identify the development of experimental malaria-associated ARDS before death, using parasitemia, breath frequency and enhanced pause (Penh), measured on the 7^th^ dpi, recognizing the progress to ARDS or not (5). In this predictive model, it is not possible to identify the development of ARDS earlier, before 7^th^ dpi, because on the 5^th^ dpi there are no significant differences in these parameters, which is possible now through SPECT/CT diagnosis. In addition, we reinforce here the results presented by Ortolan et al., regarding hemorrhages, pulmonary edema and hydrothorax and worse respiratory capacity [5]. On the other hand, ARDS-developing mice on the 7^th^ dpi showed higher parasitemia compared to those HP-developing.

Tidal volume, which is correlated with pulmonary compliance and respiratory acidosis during the development of ARDS, is critical for monitoring patients and demonstrates inadequate pulmonary ventilation and the necessity of positive end-expiratory pressure (PEEP) adjustments to reduce the risk of death in 22% of patients in intensive therapy units [47–49]. Our results showed a reduction in tidal volume in ARDS-developing mice, corroborating the difficulty in breathing capacity.

ARDS patients present an increase in inspiratory pause [50] and inspiratory time [51], due to deficiency in pulmonary gas exchange, inducing barotrauma. Also, it is observed in these patients, extending expiration time, demonstrating the decreased ventilation and promoting pulmonary collapse [46]. Penh measurement during ARDS experimental model, which evaluates lung function noninvasively and ventilation volume, demonstrates the difficulty in gas exchange and pulmonary function failure [4,5,52–54], as observed in our results. However, as these measurements cannot be correlated with pulmonary resistance, ventilation mechanism studies are necessary [55].

Imaging techniques as well as chest radiography and computed tomography have been used to ARDS diagnosis [56] and also to malaria patients, allowing the identification of diffuse bilateral opacification and other lung lesions [6,57]. However, in our murine model, we observed the predominance of diffuse unilateral opacification [5] and in lipopolysaccharide model no predisposing lesions (apex or pulmonary base) were detected [18].

Morphological analysis of lung tissues are essential for ARDS diagnosis but it is necessary that two experienced radiologists confirm the lesions, which makes the quantitative analyzes fundamental to optimize processing time and promotes greater diagnostic accuracy [58,59]. The main lesions during ARDS, in the acute phase, are characterized by intense areas of ground-glass attenuation which demonstrates low aeration and consolidation, displaying pleural effusion and increased pulmonary permeability [18,60–62]. In the late phase, it is still possible to observe the reticulation attenuation demonstrating interlobular septal thickening, due to inflammatory cells and fibrosis accumulation [56,63,64]. Our results corroborate literature, since ARDS-developing mice demonstrated reduced pulmonary aeration and increased vascular permeability.

The decrease in pulmonary aeration (> 40%) and the increase in vascular permeability are related to decreased compliance, due to a deficiency in the lung elastic properties during ARDS [26,65]. These findings are compatible with our results since the ARDS-developing mice present an increase in vascular permeability evidenced by pleural effusion reaching 1 ml of fluid in the thoracic cavity, which would complicate compliance. Our previous results showed increased pulmonary vascular permeability in malaria infection in DBA/2 model both *in vivo* and *in vitro* [4,5,38,39]. In addition, we observed that, after 14^th^ dpi, an improvement in lung aeration when compared to that on the 7^th^ dpi. Also, the baseline respiratory frequency and Penh in HP-developing mice, on the 21^st^ dpi, returned to baseline, similar to that non-infected mice [5].

Chest computed tomography shows that 85% of the patients present bilateral lesions in the frontal segment of the left lung and only 5% of them do not exhibit these characteristics, indicating a high sensitivity of this technique in ARDS lesions when compared to chest X-ray [66]. Alveolar-capillary increased permeability in these patients associated to decreased surfactant and lung inflammation induce atelectasis [65]. Pulmonary ventilation reduction is observed during ARDS, demonstrating pulmonary collapse, especially due to edema formation [67,68].

The CT is essential to prevent patients death in intensive care units. The reduction of ARDS patients mortality consists of not only hypoxia analysis, but also of PEEP, tidal volume and respiratory regulation assessment and adjustment which diminish lung collapse and overdistention, promoting protective mechanical ventilation [69,70].

The quantitative CT analysis shows that, during ARDS, deficit in gas exchange occurs with increased poorly aerated and nonaerated tissues and, decreased aerated tissues [71], in agreement with atelectasis, ground-glass opacification and consolidation attenuation areas [58], contributing to the reduction of pulmonary aeration [69]. High-resolution tomography is widely used in the measurement of pulmonary lesions and fibroproliferation in ARDS patients [72]. Segmentation of pulmonary aeration quantification during ARDS in both human and rodent models demonstrates to be an efficient tool for diagnosis, facilitating the data interpretation [24,71]. Recently, it has been described, in ARDS patients, a decrease in hyperinflated and normally aerated, reduction of poorly aerated and increase of nonaerated tissues, with values higher than 2-fold when compared to controls, demonstrating alveolar collapse and respiratory function failure [24]. Reduction in pulmonary aeration is observed in several human or animal ARDS models and proves to be a useful method for assessing atelectasis at the onset of lung injury [73–75].

The increase of nonaerated tissues is a milestone in the evaluation of pulmonary ventilation during ARDS [65], since these values can exceed 50 times those found in the voluntary controls, being CT an important tool to lung lesions identification. In addition, there is a decrease in hyperinflated and normally aerated tissues, especially on the right side of lungs [63,76]. Therefore, pulmonary aeration analysis during ARDS allows the identification of early diagnosis regarding to pulmonary oxygenation failures, which may prevent the high mortality of patients in the intensive care unit [77], including those malaria infected.

Measurement of pulmonary oxygenation is important to assess the severity of ARDS and is correlated with increased nonaerated tissues and atelectasis [73]. The degree of hypoxemia can be related to CT findings [71], in which low PaO_2_ levels are linked to increased atelectasis areas and high shunt values [78]. In previous results, it was demonstrated, in our murine model, that hypoxemia was related to macroscopic and histological findings in ARDS-developing mice [4,5].

The respiratory mechanics, mainly perfusion, is essential to understand ARDS physiopathology [79]. During ARDS developing, the pulmonary perfusion is reduced in poorly aerated areas, which enables observation of radiopharmaceutical accumulation in the lesion, probably due to the increase of vascular permeability and higher blood bioavailability containing the radiopharmaceutical [60]. However, pulmonary alterations by CT are not always correlated with SPECT, as in lung cancer, in which there is marked decrease in aeration and intense areas of ground-glass, although the tissue analyzed by SPECT had been preserved [80].

Pulmonary perfusion in ARDS patients demonstrated a marked increase in the pulmonary base due to endothelial barrier dysfunction [77]. The perfusion location, important to measure oxygenation and radiopharmaceutical decrease, occurs in poorly aerated and it is distributed randomly in areas during ARDS [81]. The study of pulmonary perfusion and ventilation is essential during ARDS, detecting severe pulmonary insufficiency in patients [70]. Therefore, we believe these tools are very important to malaria-associated ARDS early diagnosis, enabling a more efficient therapeutic follow-up and reducing these patients mortality.

## Conclusions

The application of the quantitative computed tomography technique allowed the early diagnosis in malaria-associated ARDS, concerning two methodologies for respiratory profile quantification, allowing a higher accuracy in the pulmonary oxygenation identification and also showing failures in the distribution of blood flow in ARDS-developing mice. However, the application of the SPECT/CT methodology in the early diagnosis of malaria-associated acute respiratory distress syndrome in humans could be impaired in malaria endemic areas due to the difficulty in accessing nuclear medicine equipment. Nonetheless, PET/CT methodology associated with magnetic resonance may be further used to identify the location of the inflammatory process and injuries during malaria-associated ARDS. On the other hand, for a better understanding of the pathophysiological mechanisms during ARDS, further studies should be developed.

## Supporting information

S1 Fig Summary protocol of SPECT/CT Image acquisition and processing process. Simplified representation of the methodology used in image acquisition and processing to provide the final perfusion data sets.
(TIF)

S1 Dataset (XLSX)
