# Supplementary figures and images for: Lung aeration in experimental malaria-associated acute respiratory distress syndrome by SPECT/CT analysis
Source: PLoS One. 2020 May 29;15(5):e0233864. doi: 10.1371/journal.pone.0233864 (PMC7259762; doi:10.1371/journal.pone.0233864)

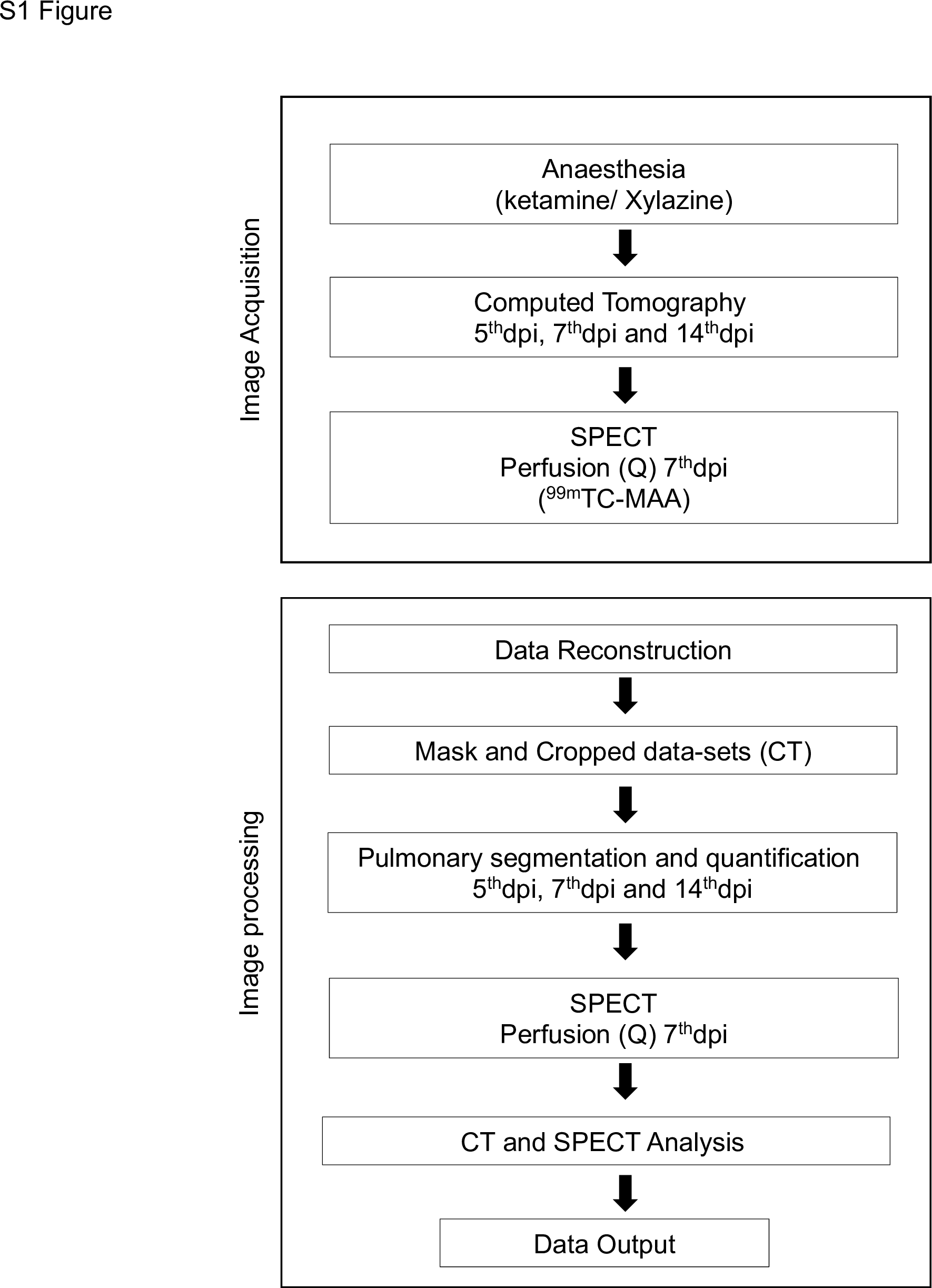

Supplement: S1 Fig — Simplified representation of the methodology used in image acquisition and processing to provide the final perfusion data sets. (TIF) [file pone.0233864.s001.tif]
